# What are the most effective interventions to improve physical performance in pre-frail and frail adults? A systematic review of randomised control trials

**DOI:** 10.1186/s12877-019-1196-x

**Published:** 2019-07-11

**Authors:** Tara Kidd, Freda Mold, Claire Jones, Emma Ream, Wendy Grosvenor, Märtha Sund-Levander, Pia Tingström, Nicola Carey

**Affiliations:** 1 School of Natural Sciences and Psychology, Liverpool John Moores University, Liverpool, UK; 2 School of Health Science, University of Surrey, Surrey, GU2 7YH UK; 3 Department of Medical and Health Sciences, Linköping University, Linköping, Sweden

**Keywords:** Frailty, Successful aging, Physical activity, Nutrition, Intervention

## Abstract

**Background:**

With life expectancy continuing to rise in the United Kingdom there is an increasing public health focus on the maintenance of physical independence among all older adults. Identifying interventions that improve physical outcomes in pre-frail and frail older adults is imperative.

**Methods:**

A systematic review of the literature 2000 to 2017 following PRISMA guidelines and registered with PROSPERO (no. CRD42016045325).

**Results:**

Ten RCT trials fulfilled selection criteria and quality appraisal. The study quality was moderate to good. Interventions included physical activity; nutrition, physical activity combined with nutrition. Interventions that incorporated one or more physical activity components significantly improved physical outcomes in pre-frail and/or frail older adults.

**Conclusions:**

Physical activity interventions are key to maintaining independence in pre-frail and frail older adults. A lack of consensus regarding the definition of frailty, and an absence of core measures to assess this means any attempt to create an optimal intervention will be impeded. This absence may ultimately impact on the ability of older and frail adults to live well and for longer in the community.

**Electronic supplementary material:**

The online version of this article (10.1186/s12877-019-1196-x) contains supplementary material, which is available to authorized users.

## Background

Frailty, a geriatric syndrome characterized by unintentional weight loss, low muscle strength, feeling of exhaustion, reduced physical activity capacity and slow walking speed [22, 34, 46], affects 4–60% adults aged ≥65 years [11] and is associated with significantly increased risk of poor physical health, hospitalization, nursing home care and mortality [18, 29, 41]. In an aging society the rapidly increasing number of frail older adults and associated rise in healthcare expenditure [19] is seen as a major challenge facing health and social care [1].

Despite growing interest in this topic a widely accepted definition and clear criteria for frailty is lacking [7]. Currently, the Cardiovascular Health Study (CHS) frailty phenotype, also known as the Fried Criteria [22], which focuses on physical phenotype, is the most widely used tool for assessing frailty status [21].

There is a growing consensus that interventions targeting the physical phenotype associated with increased risk for adverse outcomes in older adults; particularly mobility, strength, balance, nutrition and physical activity, may offer the best opportunity to prevent, delay, or reverse existing symptoms of physical frailty [3, 9]. Evidence from two recent systematic reviews identified a range of interventions, i.e. physical activity, nutrition, geriatric assessment or a blend of these delivered in primary care, community settings or at home, and found those that incorporated a physical activity component were consistently the most effective at improving frailty status, physical outcomes (e.g. body mass index, muscle mass, strength, gait speed, exhaustion, physical activity) and/or functional ability [14, 39]. However, caution must be applied when interpreting change in frailty or functional ability as a primary outcome measure, as there is still a lack of agreement regarding clinically meaningful reduction in frailty or functional ability [2, 37, 38]. In contrast, performance based physical outcome measures such as mobility, balance, body mass and activity levels, have consistently reported strong associations with future health, functional ability, and service use in older and frail adults [25, 27, 47].

Identifying effective interventions, with the potential to promote successful aging and, minimise the burden of care on health care services is therefore crucial [3]. Building on previous work, by focusing only on randomised controlled trail (RCT) interventions that specifically measure one or more physical performance outcomes, a systematic review was undertaken to explore potential preventative applications of these interventions in pre frail and frail older adults.

### Research question

“What are the most effective interventions for improving physical performance outcomes in pre-frail and frail older adults?”

## Methods

A systematic review, registered with PROSPERO (no. CRD42016045325), using evidence from 2010-March 2017 and following Preferred Reporting Items for Systematic Reviews and Meta-Analyses (PRISMA) guidelines [13] was undertaken.

### Definition of terms

1. Physical performance was defined as an observable physical outcome measure related to the frailty criteria [22] specified for this study, including gait speed, grip strength, physical activity levels, mobility, balance, muscle mass, and body mass index. Body Mass Index was used as an indicator of weight loss or gain [15].

### Search strategy

Targeted searches of Cochrane Database of Systematic Reviews, MEDLINE, EMBASE, CINAHL and PsycINFO were conducted using index/MeSH (Medical Subject Heading) and string of keyword terms, (Frail Elderly) + (early intervention) + (health care, health service, patient care). See Additional file 1 for search terms, and example search string.

### Eligibility criteria

Articles were included that comprised of:

2. RCTs reporting one or more observable measure of physical performance related to frailty criteria (e.g. gait speed, grip strength, physical activity levels, mobility, balance, muscle mass, body mass index) as this study design generally supports greater validity and causal inference [40].
3. Pre-frail or frail adult participants, aged > 65 years.
4. Peer reviewed publications, available in English.

Studies were excluded if physical performance was only measured using Activities in Daily Living (ADL) or Instrumental Activities of Daily Living (IADL) to ensure physical frailty, rather than disability was assessed. Participants were also excluded if they had dementia, psychosis/personality disorders, or were institutionally confined.

### Study selection and screening

Results were exported into EndNote X7 software (Thomas Corporation) and duplicates removed before titles and abstracts were screened in relation to the inclusion/exclusion criteria. Citations were screened by all members of the research team (NC, FM, ER, WG, MSL, PT) and checked independently by the two other reviewers (TK & CJ). All reviewers confirmed the eligibility of the identified studies. Disagreement was resolved during discussions in the author team meeting. Excluded papers including systematic reviews were scanned to identify any additional articles.

### Data extraction

Data extraction was conducted by three researchers (TK, CJ, EM) using a pre-designed data extraction form to capture details about study, data collection methods, sample, outcome measures, intervention content, duration of follow-up, analysis methods, results, intervention effectiveness and limitations. The template for intervention description and replication [TIDieR] [31], designed to improving intervention reporting, was used to record intervention content.

### Strength of evidence assessment of studies

The Cochrane Risk of Bias Tool [30] comprising seven domains: sequence generation, allocation concealment, blinding of participants and personnel, blinding of outcomes assessed, treatment of incomplete data, selective outcome reporting and other risks of bias, was used to analyse each study. The risk of bias in each subcategory was classified as high, low or unclear. Assessment of bias was conducted independently by 2 authors (TK & CJ), decisions compared and discussed to achieve consensus (Additional file 2).

## Results

**Fig. 1:**
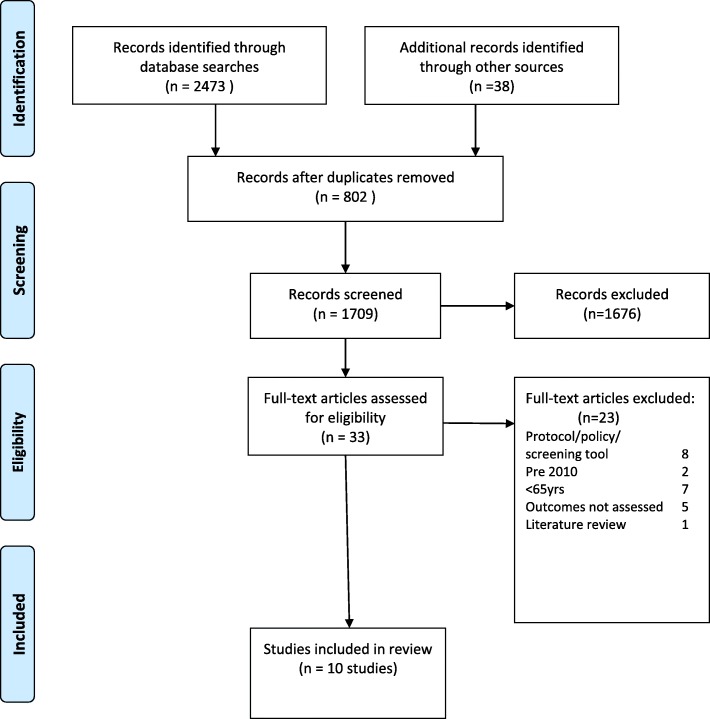
Flow diagram detailing the search process

Searches across all database and additional searches yielded *n* = 2511 results. After applying the inclusion/ exclusion criteria *n* = 33 remained. Full text articles were retrieved and on closer inspection *n* = 23 did not fulfil the review eligibility. A total of 10 articles were eligible and included in the analysis (see Fig. 1 and Table 1).

**Table 1:** Description of included studies

Author (Year)	Sample Size	Age Mean ± SD	Frailty criteria	Frailty status at baseline	Intervention	Duration and frequency	Setting	Follow up	Outcome(s) measured	Summary of findings
[6]	53 (I) 49 (C)	84 84	Gait speed of less than 0.8 m/s PASE score of less than 64 for men and 52 for women.	Mixed (NF, PF, F).	Self-administered exercise (mobility, strength, balance, and endurance training) and nutritional supplement program supervised by home helpers.	1 x daily 20 min exercise session over 4 months. 2 doses of 10 g amino acids taken over 1.5 months.	Primary care	4 months	Physical Activity (PASE) Functional status Walking time and distance Nutritional status	Significant between-group difference in maximum walking time and distance in good compliers at 4 months (*p* < 0.05). No significant intervention effects for other measures of frailty.
[8]	120 (I) 121(C)	83.4 ± 5.81 83.2 ± 5.9	≥3 CHS criteria	Frail	Individually tailored physical activity and nutrition program (protein supplements were offered to those whose BMI was less than 18.5).	10 physiotherapy sessions delivered over 12 months. Nutritional component as needed.	Primary care	3 months 12 months	Frailty (CHSC) Mobility (SPPB) Disability Service use	Significantly lower prevalence of frailty and better mobility in intervention vs. control at 12 months (*p* < 0.05). No difference on any other outcome measures.
[20]	120 (I) 121 (C)	83.4 ± 5.81 83.2 ± 5.9	≥3 CHS Criteria	Frail	Individually tailored physical activity and nutrition program (protein supplements were offered to those whose BMI was less than 18.5.)	10 physiotherapy sessions delivered over 12 months. Nutritional component duration/frequency not described.	Primary care	3 months 12 months	Fall rates Risk factors for falls (PPA) Mobility (SPPB) 4 min walk test	No difference in fall rates between groups. Improvement in risk factors for falls including quadriceps strength and sway, gait speed and mobility were reported at 12 months for the intervention group (all p < 0.05).
[24]	22 (1) 19 (C)	84.1 ± 3.0 83.9 ± 2.8	≥10s to perform a rapid-gait test. Unable to stand up 5x from seated position). Self-reported exhaustion.	Frail	Physical Activity (Functional balance and lower body strength based exercise)	2x weekly group classes of 45 min duration over a 12 week period.	Primary care	12 weeks 36 weeks	Balance (semi-tandem and tandem stands and single leg balance) Physical function (MTUG) Gait speed Lower body Strength ADL (measured using Barthel Index)	Significant sustained improvements in balance, mobility and physical function for intervention group at 36 weeks (*p* < 0.05)
[35]	196 (I) 50 (C)	69.7 ± 4.23 70.15 ± .2.0	CHS frailty criteria	Mixed (pre-frail to frail)	Physical activity Nutrition Cognitive Training Or Combination	Physical activity 90mins 2x weekly group sessions for 12 wks. Given daily exercises to do at home Nutritional supplements (iron, B6, B12, calcium, vitamin D) daily for 24 wks. Cognitive training 2 h weekly sessions for first 12 weeks, then 2 weekly booster sessions for remaining 12 wks.	Primary care	3,6,12 months	BMI Physical activity levels Knee extension strength Frailty Gait speed Functional ability (ADL) Hospitalisation Falls	Frailty scores and status were improved in all groups including control at 12 months: Significantly greater improvements for the intervention groups (combination, physical activity, nutrition and cognitive gps respectively). Strength was significantly improved in the physical activity, cognitive training and combination gps. Physical activity was significantly improved in the nutritional gp. Gait speed improvements were reported for physical activity group only. (all p < 0.05). Improvements were sustained at 12 months.
[48]	175 (I) 142 (C)	83.1 ± 5.8 83 ± 6.3	CGA	Mixed (NF, PF, F).	Physical activity (Individualised mobility plan following surgery for hip fracture)	Day 1post operation, patients were mobilised with assistance. Day 2–4 based on individual progression Physical assessment on day 4 for 24 h. Questionnaire data assessed day 5.	Secondary care	5 days following surgery	Upright time (standing, and walking) Need for assistance (CAS) Mobility (SPPB)	Intervention group had significantly more upright times, higher number of upright events, and better Short Physical Performance Battery scores than the control participants (p < 0.05). No difference on need for assistance(*p* > 0.05)
[49]	198 (I) 199 (C)	83.4 ± 5.4 83.2 ± 6.4	CGA	Mixed (NF, PF, F)	Physical activity (Individualised mobility plan plus home exercise plan post discharge)	Daily evaluations in hospital and at home rehabilitation arranged 2 weeks post hospital discharge. Assessments at 4 and 12 months.	Secondary care	4 and 12 months	GAITRite (Gait speed Step length Cadence Rhythm Postural control Gait asymmetry). Nottingham E-ADL	Significant improvements in the 4 min gait speed test at 4 and 12 months and gait characteristics including pace, rhythm, postural control, and less gait asymmetry at both time points for the intervention gp. A significantly higher proportion of participants in the intervention group were able to walk independently at 12 mths (*p* < 0.05).
[50]	31 (I) 34 (C)	78 ± 1 81 ± 1	> 1 CHS Frailty Criteria	Mixed (prefrail and frail)	Nutrition	Intervention - 2x drinks daily for 24 weeks2 containing 15 g protein, 7.1 g lactose, 0.5 g fat, and 0.4 g calcium). Control – 2x Placebo drinks daily for 24 weeks (no protein, 7.1 g lactose, 0.4 calcium)	Primary care	24 weeks	Body composition and bone mineral density Physical performance (SPPB) Strength (Leg press and extensions, hand grip strength)	No significant differences reported on body composition /bone mineral density parameters or strength outcomes. Physical performance increased for the intervention group at 24 weeks. Specifically, the intervention group were significantly faster at the chair rise test (p < 0.05).
[51]	76 (I) 76 (C)	79.1 ± 6.4 80.7 ± 6.0	BBS ≤49/56 ≤ 1 falls in past 6 months.	Mixed (prefrail and frail)	Physical activity (Tai chi vs standard physiotherapy)	2 × 60 min weekly group sessions for 15 weeks.	Secondary	15 weeks	Falls Balance (BBS Foam and dome test)	No significant difference between groups on any outcome measure.
[52]	77 (WN) 70 (W) 75 (C)	76.3 ± 5.9 75.8 ± 5.2 75.7 ± 6.5	CHS Frailty Criteria	Mixed (NF, PF, F).	Physical activity (walking) plus/minus nutrition.	Walking and nutrition (WN). Daily nutrition supplements containing protein (10 g), vitamin D (12.5 mg), calcium (300 g), plus daily walking program every day lasting for 6 months. Steps to be increased 10% each month. (W) Daily walking for 6 months (C) No intervention	Primary	6 months	Walking Biochemical (Anabolic hormones) SMI	In both the W/N and W groups, the average daily steps were significantly increased compared to control (*p* < .01). Significant improvements in interventions groups on skeletal muscle mass and anabolic hormone production (*p* < .05) compared to control.

### Study characteristics

Of the 10 included studies 4 were physical activity interventions [24, 48, 49, 51], 5 physical activity plus nutrition [6, 8, 20, 35, 52], and 1 nutrition only interventions [50]. Methodological quality ranged from adequate (*n* = 3) to excellent (*n* = 7) (see Additional file 2).

Multiple outcomes were assessed both within and across studies, with mobility or its components the most commonly reported outcome [6, 8, 20, 24, 35, 48, 49, 52], followed by physical capacity [24, 35, 50, 52], service use and mortality [8], and falls [20, 51].

Six studies were based in a primary care setting including participants home [6, 8, 20, 24, 35, 52], 3 in secondary (hospital) care [48, 49, 51], with one unclassified setting [50].

Studies were predominantly delivered face-to-face on an individual basis [6, 8, 20, 24, 48, 49], with 1 utilising group delivery [51], and 2 remote delivery methods [50, 52]. Follow-up ranged from 1 week-24 months, with most reporting data at 3, 6 or 12 months. Sample sizes ranged from 41 to 397, and studies were conducted in a wide variety of countries, the most commonly reported was Australia (*n* = 2), followed by France, Norway, Sweden, the Netherlands, Canada, Singapore, Japan, and Barcelona (all *n* = 1). No studies originated from the UK.

All 10 articles included a measure of physical frailty. Frailty was not clearly defined, which was reflected in the heterogeneity of assessment measures. Validated measures used included the CHS frailty phenotype or Fried criteria [22], Comprehensive Geriatric Assessment, and Physical Activity Scale for Elders.

### Physical activity interventions

#### Primary care setting

Giné-Garriga et al. [24] incorporated group based exercise focusing on balance, and upper and lower body strength, along with function focused activities designed to mimic everyday tasks. The intervention comprised of twice weekly 45 min classes over 12 weeks. Significant improvements were reported in the primary outcome measures of the Barthel Index, rapid gait test, and stand up test, which were maintained at 36 weeks (all *p* < 0.05) [24]. Significant improvements were also reported for the intervention group in secondary outcomes including balance, gait speed, and lower body strength (*p* < 0.05).

#### Secondary care settings

A total of 3 physical activity intervention studies were based in hospital settings. One focused on falls prevention [51], while 2 examined effects of a physical function intervention on mobility outcomes following a surgical procedure [48, 49]. The falls prevention program intervention compared Tai Chi with conventional physical therapy for frail older adults at risk of falls [51]. Both groups received twice weekly 60 min sessions for 15 weeks via a group setting for Tai Chi, and individually for the conventional treatment. Both groups improved but the Tai Chi group did not reduce fall frequency significantly better than conventional treatment, even though a trend emerged for lower fall rates in the Tai Chi group. Given that both the Tai Chi and conventional treatment groups had over 40% drop out rates across the duration of the intervention (*n* = 29 and 35 respectively), any effects may be underestimated due to a lack of statistical power.

Two studies reported on the Trondheim hip fracture RCT [48, 49] which examined physical activity and mobility in the immediate post-surgery days on a geriatric ward compared with a conventional post-surgery ward [48]; assessing gait characteristics at 4 and 12 months post intervention ([49]). The post-operative mobilisation plan comprised: mobilisation 24 h following surgery; mobilisation goals based on initial performance, training and practising activities related to daily living; strength training was also included if required, and ward routines designed to prohibit long periods of sitting or lying.

Taraldsen et al. [48] found that those receiving the intervention had significantly greater upright time (*p* = 0.016), number of upright events (*p* = 0.005), and better physical performance than conventionally treated counterparts four days following surgery (*p* = 0.002) [49] found that significantly more patients could perform the 4 min gait speed test at 4 (*p* = 0.049), and 12 months (*p* = 0.005) than conventionally treated patients, and overall had better gait characteristics including pace (*p* = 0.001), rhythm (*p* = 0.019), postural control (*p* = 0.027), and less gait asymmetry (*p* = 0.004) at 12 months. A significantly higher proportion of participants in the intervention group were able to walk independently (*p* = 0.006), had better outdoor mobility (*p* = 0.015), and greater independence when using public transportation at 12 months compared to controls (*p* = 0.040). Length of stay was slightly longer for the intervention group (12.6 vs 11 days); however, the intervention was found to have an 88% probability of being both less costly and more effective than orthopaedic care in the long run.

### Physical activity plus nutrition

#### Primary care settings

Of the 5 studies examining physical activity plus nutrition in a primary care setting, Fairhall et al. [20] and Cameron et al. [8] report data taken from the same RCT of a multifactorial intervention designed to target frailty characteristics including nutritional assessment, physiotherapy and medical management to reduce fall rates. Ten physiotherapy sessions, delivered over a 12 month period, focussed on strength and balance, with high energy, high protein supplements offered to those whose BMI was less than 18.5 (*n* = 60, 50% of sample).

In total, 25 participants (21%) were recommended vitamin D supplements. Adherence to the nutritional intervention ranged between 26 to 50%. Fairhall et al. [20] found no difference in fall rates between the intervention and conventional treatment groups (*p* > 0.05). Cameron et al. [8] found significant reductions in frailty status at 12 but not 3 month follow up (*p* = 0.02). Improvement in physical performance including strength and gait speed were reported at 12 months (all *p* < 0.001). No differences were found on mortality, hospital admissions, permanent admissions to nursing care facilities, or quality of life outcomes (*p* > 0.05).

Bonnefoy et al. [6] devised an intervention around existing home help services which combined a self-administered exercise program, (participants were prescribed exercises by a physiotherapist, received a booklet explaining how to perform exercises, a poster showing pictures of the exercises, and how to fill in a compliance diary) alongside a 10 g amino-acid supplementation to be taken under the supervision of the home help. It was expected that the visitation of the home help would also prevent sedentariness as they were trained to encourage physical activity. Overall adherence was poor to the exercise and nutritional components, with only 44% (*n* = 23/53) of participants being fully compliant. The results of the study suggest limited impact of exercise and nutrition on markers of frailty, including body composition indicators, mobility, or activities in daily living.

Ng et al. [35] randomised participants with a mean age of 70 years to physical exercise, nutrition, cognitive training or combination treatment program for 6 months to examine the impact on frailty characteristics, with specific focus on mobility and strength performance outcomes. Frailty was assessed and included participants who were classified as pre-frail or frail, though no stratified analyses were conducted. The exercise group (*n* = 48) received a program tailored to their specific ability in classes for 90mins twice weekly for 12 weeks which focused on strength and balance. This was followed by a 12 weeks of home exercises. The nutritional group (*n* = 49) received supplements of iron and folate, vitamins B6 and B12, calcium and vitamin D for 24 weeks. Cognitive training (*n* = 50) involved 2 h weekly sessions for 12 weeks of cognitive-enhancing activities designed to stimulate short-term memory, and enhance attention, information-processing skills, reasoning and problem solving abilities. The following 12 weeks included fortnightly booster sessions. The combination group (n = 49) experienced all treatments. Frailty scores were significantly reduced at 6 and 12 months in all groups (all *p* < 0.05), with combination group reporting the greatest reduction (mean change = 5.00), followed by physical therapy (mean change = 4.05), nutrition (mean change = 2.98) and cognitive therapy (mean change = 2.89). However, there was no clear statistical difference between treatment groups on improving physical performance. Lower body strength improved in the combination, physical activity, and cognition groups (*p* = 0.009); gait speed improved in the physical activity group only (*p* < 0.001); overall physical activity improved in the nutrition group (*p* = 0.038).

The final study by Yamada et al. [52] was the only study that delivered the intervention by remote delivery methods. This was a pedometer based walking program and nutritional supplement (protein and vitamin D) delivered over a 6 month period. Participants were randomly assigned to a walking group (*n* = 71), walking plus nutrition (*n* = 79), or control group (*n* = 77). Adherence levels were very high, with 80% adherence reported for the nutritional component, and 100% for the walking initiative. Muscle mass and biochemical outcomes were assessed. Both the walking and walking plus nutrition groups were successful in improving biochemical outcomes associated with improved muscle mass (IGF-1, 25(OH)D) and skeletal muscle mass index compared with the control group (*p* < 0.05), but only the walking plus nutrition group had significantly greater improvements in Dehydroepiandrosterone-Sulfate (DHEA-S) (p < 0.05). The number of daily steps also increased significantly in both groups, with an increase from 4471 to 6067 steps in the walking plus nutrition group, and an increase from 3795 to 5394 steps in the walking group. Sub-group analysis was conducted separately for non-frail and frail participants; however the sample size for this analysis was small with only 31 frail vs 46 non-frail in the walking plus nutrition group, 15 frail vs 55 non-frail in the nutrition only group, 25 frail vs 50 non-frail in the control group; however, cautious interpretation suggests that both frail and non-frail participants benefited in improved physical performance from the intervention in comparison to the control group.

### Nutrition

#### Primary care setting

Tieland et al. [50] recruited pre-frail and frail participants to receive twice daily protein supplements vs a placebo over a 24 week period; however despite high reports of adherence, there was no difference in any biochemical measure, skeletal muscle mass, or in hand or lower body muscle strength compared to the placebo group. Only physical performance, (a composite score of gait, balance and chair rise test) significantly improved in the protein group at 24 weeks (*p* = 0.02). The authors report that the increase in physical performance was of substantial clinical relevance and translates to a 30% relative risk reduction for disability and a reduced risk for institutionalization and mortality.

## Discussion

This review found interventions including one or more physical activity components were successful at improving physical performance in pre-frail and frail older adults, with some evidence to suggest deterioration was ameliorated up to 12 months post-intervention [48, 49]. Contrary to previous work, we found no clear evidence to support the superiority of multi-domain interventions over simple interventions [14]. Given the increasing concerns regarding the projected rise in older people in relation to future service provision this review is timely, and of significant importance.

Several factors related to intervention success: firstly, interventions targeted to improve physical condition, e.g. resistance training to build muscle mass and strength, and a clearly defined outcome (e.g. upper or lower body strength) reported significant improvements [24, 48]. Secondly, interventions combining resistance and balance training were most successful in treating physical symptoms associated with frailty, reducing falls, and maintaining health benefits [20, 25, 35, 48, 49]. Combining different types of physical exercise may therefore support maximum impact on all physical performance components associated with frailty i.e. mobility, balance, body mass, levels of activity.

Thirdly, supervised interventions across primary and secondary care reported improved physical performance [8, 20, 24, 35, 48–50]. Supervision of physical exercise may be an essential element for success for a number of reasons: importantly, supervision promotes exercise regimen adherence, which is critical to experiencing beneficial effects. Relatedly, frail individuals are likely to have concerns about their ability and falling which supervision and support can help overcome [4, 28]. A significant proportion of frail older adults are cognitively impaired, which may also affect exercise regimen adherence. While this review did not examine interventions in people with cognitive decline, studies report promising results for supervised physical activity interventions in people with dementia [26], suggesting that benefits of supervised physical activity can be applied across the frailty spectrum.

One community-led supervised physical activity interventions also demonstrated successful results [24]. Community-based exercise, such as Tai Chi, has previously been shown to reduce hospital admissions, falls, and admission to long-term healthcare [5, 23]. This is particularly relevant in the UK where government policy priority emphasises the need to move the provision of non-emergency healthcare from acute to primary care [16].

Our results tentatively suggest the potential for home based individualised physical training programs for older adults [52]. Evidence suggests that interventions could be delivered remotely via the telephone [36], mobile applications [44] or virtual reality and gaming technology [45]. Initial results appear promising with reported gait and balance improvements, but these approaches are yet to be tested in frail older adults [45]. Home-based programs are more accessible, and potentially cost effective, eliminating transportation barriers for many frail adults, enabling them to be active, live well and for longer in their local community [16, 43].

Given that reducing falls, service use and/ or admission to a permanent care facility is a core component of current health care policy [16, 19] the results from this review are encouraging. Targeted interventions to improve balance and muscle strength have been shown to reduce falls risk, and subsequent hospital admissions [32, 42]. Although few of the reviewed studies reported on falls, service use or placements [48, 49], the limited evidence indicated that targeted physical interventions were associated with improvements in these outcomes.

While the benefits of nutritional intervention cannot be determined by one study, the wider literature suggest the potential benefits of targeting those who are malnourished [10]. Malnutrition is associated with poor health outcomes including reduced functional status, decreased muscle mass, higher risk of permanent care placement and mortality [12, 33]. This provides some support for international guidelines suggesting that nutritional interventions should be given as a preventative measure to older people at risk of malnutrition [17], for example, frail pre-surgical patients to enhance recovery. Hospital based pre-assessment clinics would be ideal setting to incorporate mandatory screening for malnutrition and delivering this targeted type of intervention.

### Limitations

Reviewed interventions were designed to achieve rapid improvements in physical performance over relatively short time periods; however, disparity between studies meant the intensity and frequency of intervention delivery needed to achieve and maintain these physical benefits was unclear. Also, information about participants’ activity levels at follow-up was not reported and it is not clear if the reported physical benefits were due entirely to the intervention, or if there had been some sustained behaviour change in physical activity.

On reflection, the search strategy for this review may have been too broad. Varied definitions of frailty were incorporated across the studies making meaningful comparisons difficult. Information was often limited regarding the proportion of participants who fulfilled pre-frail, or frail physical criteria within studies. Small sample sizes meant that pre-frail and frail participants were often grouped together for analyses purposes, so it was not possible to ascertain whether changes in physical performance translated into outcomes that were clinically meaningful. Consequently, we were unable to consider preventative vs. targeted treatment effects in these groups. Correspondingly, there was a wide range of outcome measures used across studies which perhaps reflects the lack of clarity over what it means to be identified as frail.

## Conclusion

This review has systematically explored the effectiveness of interventions to improve physical performance in pre-frail and frail adults. The small number of RCT available to include suggests a significant gap in the research literature. Relatedly, given the UK government’s commitment to improve health outcomes by 2020, it was surprising no eligible UK studies were found. Despite this, the results tentatively suggest that tailored, supervised, physical activity interventions are effective at improving physical performance components associated with frailty in both primary and secondary care settings. However, until there is an agreed definition for frailty and a core set of measures to assess this, any attempt to create an optimal validated intervention will be impeded. This absence may ultimately impact on the ability of older and frail adults to live well and for longer in the community.

## Additional files

Additional file 1: Example search string. (DOCX 22 kb)

Additional file 2: Risk of Bias. (DOCX 13 kb)

## Data Availability

Data sharing is not applicable to this article as no datasets were generated or analysed during the current study.

## Abbreviations

Ab: Abstract word
ADL: Activities of Daily Living
BBS: Berg Balance Scale
BMI: Body Mass Index
CAS: Cumulated ambulation score
CGA: Comprehensive geriatric assessment
CHSC: Cardiovascular Health Study Criteria
DHEA-S: Dehydroepiandrosterone-Sulfate
F: Frail
IADL: Instrumental Activities of Daily Living
LOS: Length of stay
MH: Main index/ MeSH term
MNA: Mini nutritional assessment
MTUG: The modified timed up and go test
NF: Non frail
PASE: Physical activity Scale for Elders
PF: Pre-frail
PI: Post intervention
PROSPERO: International prospective register of systematic reviews
PRSIMA: Preferred Reporting Items for Systematic Reviews and Meta-Analyses
PT: Publication Type
RCT: Randomised Controlled Trial
SMI: Skeletal muscle mass index
SPPB: Short physical performance battery
SU: Subject Heading
Ti: Title word
UK: United Kingdom
