# Supplementary material for: What are the most effective interventions to improve physical performance in pre-frail and frail adults? A systematic review of randomised control trials
Source: BMC Geriatr. 2019 Jul 11;19:184. doi: 10.1186/s12877-019-1196-x (PMC6622112; doi:10.1186/s12877-019-1196-x)
Supplement: Supplementary file 1 — Example search string. (DOCX 22 kb) [file 12877_2019_1196_MOESM1_ESM.docx]

**Additional File 1: Example search string**

| **Database:** Medline  **Platform:** EBSCO  **Limits:** Date of Publication: 20100101-20161231; Abstract Available; English Language; Human  **Search modes** - Boolean/Phrase | |
| --- | --- |
| 1  2  3  4  5  6  7  8  9  10  11  12  13  14  15  16  17  18  19  20  21  22  23  24  25  26  27  28  29  30  31  32  33  34  35  36  37  38  39  40  41  42  43  44  45  46  47  48  49  50  51  52  53  54  55  56  57  58  59  60  61  62 | (MH "Frail Elderly")  (MH "Geriatrics")  (MH "Health Services for the Aged")  "frailty syndrome*".AB.TI  ((frail old*) AND (people OR adult* OR person)).AB.TI  "frail elder*".AB.TI  "frail old* adult*" OR "frail old* people" OR "frail old* person*".AB.TI  OR/1-7  (MH "Early Medical Intervention")  early AND (detect* OR intervention* OR recognition OR treatment*)). AB.TI  initial AND (detect* OR intervention* OR recognition OR treatment*)).AB.TI  prompt AND (detect* OR intervention* OR recognition OR treatment*)).AB.TI  rapid AND (detect* OR intervention* OR recognition OR treatment*)).AB.TI  timely AND (detect* OR intervention* OR recognition OR treatment*)).AB.TI  "timely intervention*" OR TI "timely intervention*".AB.TI  "prompt intervention*" OR TI "prompt intervention*".AB.TI  "initial intervention*" OR TI "Initial intervention*".AB.TI  "rapid intervention*" OR TI "rapid intervention*".AB.TI  OR/9-18  *PT randomized controlled trial*  *PT controlled clinical trial*  *SU randomized controlled trials*  *SU random allocation OR (MH "Random Allocation")*  *SU double blind method OR (MH "Double-Blind Method")*  *SU single blind method OR (MH "Single-Blind Method")*  *OR/20-25*  *PT clinical trial*  *AB (clin* N25 trial*) OR TI (clin* N25 trial*)*  *AB ( ((singl* or doubl* or trebl* or tripl*) N25 (blind* or mask) )*  *TI ( ((singl* or doubl* or trebl* or tripl*) N25 (blind* or mask) )*  *SU placebos*  *AB placebo* OR TI placebo**  *AB random* OR TI random**  *AB randomi#ed control$ trial$ OR TI random**  *AB randomi#ed OR TI randomi#ed*  *AB randomly OR TI randomly*  *trial.AB.TI*  *SU research design OR (MH "Research Design")*  *OR/27-38*  *S26 OR S39*  *SU comparative study*  *SU evaluation*  *AB ( evaluation (method* OR research OR model* OR process*) )*  *TI ( evaluation (method* OR research OR model* OR process*) )*  *SU follow up studies*  *SU prospective studies*  *(MH "Prospective Studies")*  *(MH "Follow-Up Studies")*  *TI (control* or prospectiv* or volunteer*)*  *(MH "Cross-Over Studies") OR SU cross over studies*  OR/41-50  (MH "Delivery of Health Care")  (MH "Delivery of Health Care, Integrated")  (MH "Continuity of Patient Care")  (MH "Comprehensive Health Care")  (MH "Health Services Needs and Demand")  (MH "Health Services Accessibility")  OR/52-57  8 AND 19  8 AND 58  59 OR 60  *40 OR 51 AND 61* |
