# Supplementary material for: What are the most effective interventions to improve physical performance in pre-frail and frail adults? A systematic review of randomised control trials
Source: BMC Geriatr. 2019 Jul 11;19:184. doi: 10.1186/s12877-019-1196-x (PMC6622112; doi:10.1186/s12877-019-1196-x)
Supplement: Supplementary file 2 — Risk of Bias. (DOCX 13 kb) [file 12877_2019_1196_MOESM2_ESM.docx]

Additional file 2: Risk of Bias

| **Column1** | **Column2** | **Column3** | **Column4** | **Column5** | **Column6** | **Column7** | **Column8** |
| --- | --- | --- | --- | --- | --- | --- | --- |
|  | **Random sequence generation** | **Allocation concealment** | **Blinding** | **Incomplete outcome data addressed** | **Selective outcome reporting** | **Other bias** | **Decision** |
|  |  |  |  |  |  |  |  |
| **Bonnefoy et al., 2012** | Unclear | Low | High | Low | Low | High | Moderate to high |
| **Cameron et al., 2013** | Low | Low | High | Low | Low | High | Low to moderate |
| **Fairhall et al., 2013** | Low | Unclear | Unclear | Low | Low | Unclear | Low to moderate |
| **Giné-Garriga et al.2010** | Unclear | Unclear | Unclear | Low | Low | Unclear | Unclear |
| **Ng et al., 2015** | Unclear | Unclear | High | Unclear | Low | High | Moderate to high |
| **Taraldsen et al. 2013** | Low | Low | High | Low | Low | Unclear | Low to moderate risk |
| **Thingstad et al., 2016** | Low | Low | Low | Unclear | Low | Unclear | Low risk |
| **Tieland et al., 2012** | Low | Low | Low | High | Low | Unclear | Low to moderate risk |
| **Tousignant et al., 2012** | Unclear | Unclear | Unclear | High | Unclear | Unclear | Moderate risk |
| **Yamada et al., 2015** | Unclear | Unclear | Unclear | High | Unclear | Unclear | Unclear |
